# Retraction notice: Host protein CD63 promotes viral RNA replication by interacting with human astrovirus non-structural protein nsP1a/4

**DOI:** 10.1099/jgv.0.001372

**Published:** 2020-08-03

**Authors:** The Microbiology Society

**Affiliations:** ^1^​ Microbiology Society, 14–16 Meredith Street, London, UK

The article ‘Host protein CD63 promotes viral RNA replication by interacting with human astrovirus non-structural protein nsP1a/4’ which was published in the *Journal of General Virology* in February 2019 has been retracted following findings reported by a reader of the journal. The immunoblots shown in Figs 3a, b, 4a, b and 6 are believed to have been manipulated and to contain duplicated images.

*Journal of General Virology* has attempted to contact the authors but to date has not received a response. All authors have been notified of this decision.

